# Development of Cycloaliphatic Epoxy-POSS Nanocomposite Matrices with Enhanced Resistance to Atomic Oxygen

**DOI:** 10.3390/molecules25071483

**Published:** 2020-03-25

**Authors:** Mayra Y. Rivera Lopez, Javier Martin Lambas, Jonathan P. Stacey, Sachithya Gamage, Agnieszka Suliga, Andrew Viquerat, Fabrizio Scarpa, Ian Hamerton

**Affiliations:** 1 Bristol Composites Institute (ACCIS), Department of Aerospace Engineering, School of Civil, Aerospace, and Mechanical Engineering, Queen’s Building, University of Bristol, University Walk, Bristol BS8 1TR, UKjm14106@bristol.ac.uk (J.M.L.); jonathan.stacey@bristol.ac.uk (J.P.S.); f.scarpa@bristol.ac.uk (F.S.); 2 Department of Mechanical Engineering Sciences, Faculty of Engineering and Physical Sciences, University of Surrey, Guildford, Surrey GU2 7XH, UK; agnieszka.suliga@esa.int (A.S.); a.viquerat@surrey.ac.uk (A.V.)

**Keywords:** atomic oxygen, cycloaliphatic epoxy resins, nanocomposites, POSS, space composites, thermoset polymers, ultra-thin laminates

## Abstract

The preparation of ultra-thin CFRP laminates, which incorporate a cycloaliphatic epoxy resin reinforced with polyhedral oligomeric silsesquioxane (POSS) reagent nanofiller, using out-of-autoclave procedure is reported. The influence of the amount of POSS within the laminate on the mechanical properties and surface roughness of the laminates is analysed before and after exposure to atomic oxygen (AO) to simulate the effects of low Earth orbit (LEO). The addition of 5 wt% POSS to the base epoxy leads to an increase in both flexural strength and modulus, but these values begin to fall as the POSS content rises, possibly due to issues with agglomeration. The addition of POSS offers improved resistance against AO degradation with the laminates containing 20 wt% POSS demonstrating the lowest erosion yield (1.67 × 10^−24^ cm^2^/atom) after the equivalent of a period of 12 months in a simulated LEO environment. Exposure to AO promotes the formation of a silicon-rich coating layer on the surface of the laminate, which in turn reduces roughness and increases stiffness, as evidenced by measurements of flexural properties and spectral data after exposure.

## 1. Introduction

The adoption of carbon fibre reinforced plastics (CFRP) is increasingly widespread in different engineering sectors such as aerospace, wind turbines, construction, and automotive, due to the high strength-to-mass ratio that they offer [1]. Consequently, CFRP materials offer very promising behaviour compared to metals, as they can achieve higher specific stiffness coupled with high thermal stability [2]. The ongoing drive for the exploration and economic exploitation of space has led to a growing interest for the development of novel lightweight materials which are affordable to launch and may withstand the extreme conditions present in the space environment. Owing to their remarkable specific mechanical properties and their flexibility to handle multi-functional requirements, CFRP materials have become increasingly popular over the past years for use in spacecraft structures [3]. As a result, these materials have been considered for past, present, and future missions, such as on the Space Telescope design, cryogenic tanks, advanced communications systems, Space Tug, Space Station, and lightweight satellites [4].

Owing to their multi-functionality, deployable structures have become more effective in the application of booms, antennas, parabolic reflectors, mirrors, solar sails, masts, and deployable solar arrays [5]. Examples of these applications are included as Supplementary Information (SI), Figure S1. These methods offer the option to minimize their volume and be compacted for storage prior to launching, then they achieve full deployment after being set to a specific position or altitude [6,7,8,9]. A significant mass decrement with the same stiffness level, as well as flexibility, are essential properties for the materials used in these applications, and for these reasons ultra-thin CFRP laminates are increasingly used. Additionally, the lower coefficient of thermal expansion (CTE) displayed by CFRP when exposed to solar radiation, compared with metal alloys, reduces the strain observed leading to lower residual stresses, thus mitigating undesirable effects seen during deployment [6]. Once deployed, CFRP structures are capable of absorbing energy through buckling, interlaminar failure, debonding between the fibre-matrix configuration, matrix deformation, or by the presence of cracks, fibre pull-out, friction, and fibre breakage [1].

However, a significant concern for composites design is the cost of the manufacturing process, the sourcing of raw materials, and the labour cost associated with the layup. Current costs of production for CFRP-based materials for aerospace applications are typically in the range of £6.38/kg, some twenty times higher than the cost of steel (£0.30/kg) or four times higher than the cost of aluminium (£1.36/kg) [1]. Furthermore, after manufacture, the launch cost to deploy a satellite structure into Low Earth Orbit (LEO) is around £15,650/kg [10]. It may be difficult to effect significant reductions in the initial cost of investment using current manufacturing methods (e.g., automated fibre layup and autoclave curing) without a paradigm shift in manufacturing technology, but increasing the longevity of the CFRP structures (and thus the lifespan of missions) is key to making the process more economical. These environmental conditions during operation can lead to the deterioration of the material properties and the accelerated degradation of spacecraft structures as they consist of photochemical damage arising from vacuum ultra-violet (VUV) radiation, thermal cycling, high velocity micrometeoroid impact, damage produced by space debris [11], and most importantly for space applications, exposure to atomic oxygen [12].

Atomic oxygen (AO) is typically found in LEO at 160 to 2000 km, the most populated altitude range above the Earth. AO is formed due to the photodissociation of diatomic oxygen molecules from the Earth’s residual atmosphere into high energy radicals by the action of UV radiation from the Sun. AO is the predominant cause of erosion in satellite structures in LEO [13] and polymeric materials are particularly susceptible to degradation when exposed to it. AO is an extremely strong oxidizing agent (possessing sufficient energy to break covalent bonds on exposed polymer surfaces) and leads to the formation of volatile oxidation products which results in the erosion of the exposed material [14]. As a consequence, it may pose serious thermal, structural, and optical complications to vulnerable materials on the exterior of spacecraft. A common approach, which has been utilised in the past to protect the surface of polymeric space structures from the effects of incidental AO is to employ protective metallic foil or metallised polymer coatings, but this introduces more weight and an additional manufacturing step into the process. More recently, the integration of inorganic silicon in the form of commercial coatings has been used: this inorganic layer has a remarkably high bond strength and oxophilicity, hence shielding the polymeric material that it covers [15]. Nevertheless, silica is an extremely brittle material and surface defects, such as scratches and cracks in the protective coating caused by problems such as the impact from micrometeroids, the deployment process, and thermal cycling, may lead to the formation of cavities that expose the underlying virgin material to the hazardous space environment [3]. This can be extremely damaging as AO radicals may undercut the silica layer and become trapped between the protective coating and the polymeric surface, leading to a rapid localised oxidation of the enclosed organic material [16].

The use of polyhedral oligomeric silsesquioxane (POSS) reagents is increasingly considered as a substitute to silicon-based protective coatings. POSS molecules are cage-like structures with a RSiO_1:5_ empirical formula, where R may be a hydrogen atom, an organic residue, or a functional group, and which have a nanoscopic diameter of approximately 1–3 nm [17]. These nanostructures consist of an inorganic silica core which may attach to a range of different functional organic groups to act as a reinforcing filler in polymeric matrix materials, resulting in a hybrid organic–inorganic character. POSS molecules are classified according to the number of reactive organic groups attached to their core cage ranging from monofunctional POSS incorporating one reactive group (and forming pendants on polymer chains) to octafunctional POSS with eight reactive groups [18]. The POSS species form a silica-rich layer similar to the silicon protective coatings upon exposure to AO. Nevertheless, when the protective layer fails and exposes the underlying material to the AO environment, the POSS nanoparticles, that are dispersed and bound to the polymeric matrix, react with the incidental AO, thus reforming the layer of passivating silica [3]. POSS-reinforced polymeric materials therefore exhibit self-healing capabilities and are able to overcome the main complication associated with other silicon-based alternatives. Additionally, numerous research studies [19,20,21] have demonstrated that the incorporation of POSS cages within polymeric matrices on a molecular level can lead to significant improvements in the material properties of these polymers, including increases in glass transition temperature (T_g_), reduction in moisture absorption, and mechanical response. In this work, an octafunctional POSS reagent (EP0409 glycidyl POSS) was selected with glycidyl ether (epoxy) groups at the apices. A diagram of the structural formula of glycidyl POSS is shown in Table 1.

CFRP structures often incorporate aerospace-qualified epoxy resins for spacecraft structures due to their high T_g_ values and high thermal stability (e.g., in this work, MTM44-1, which we have selected as a benchmark, contains tetraglycidyldiaminodiphenylmethane (TGDDM) cured with 4,4′-methylenebis(2,6-diethylaniline) and 4,4′-methylenebis(2-isopropyl-6-methylaniline)). Nevertheless, these materials with a high aromatic content are extremely susceptible to degradation when exposed to VUV radiation in the LEO environment. In contrast, the chemical structure of cycloaliphatic epoxy resins incorporates fully saturated carbocyclic rings which offer exceptional ultraviolet blocking effects for VUV stability. While cycloaliphatic epoxies do exist for use in space applications (notably those based on 3,4-epoxycyclohexylmethyl-3′,4′-epoxycyclohexane carboxylate, ECC), these are more expensive than the more commonly used diglycidylether- or tetraglycidylamine-based monomers. The ECC monomer is a low viscosity material (400 mPas at 25 °C) but is very brittle when cured, thus requiring toughening for engineering use. The cycloaliphatic epoxy resin system featured in this work has previously been studied by Suliga [22] and is reported by Suliga et al. [23], but the measurements undertaken were predominantly physical, rather than mechanical, in nature. In the present work, we have produced ultra-thin flat laminates comprising the same cycloaliphatic epoxy resin system, reinforced with a wider range of POSS loadings, in order to report more detailed mechanical properties and assess the AO stability.

## 2. Experimental Methods

### 2.1. Materials

The CY184 epoxy resin (EEW 144–172 g/eq) and Aradur 2954 cycloaliphatic amine curing agent were purchased from Huntsman Advanced Materials (Basel, Switzerland); the EP0409 glycidyl POSS was purchased from Hybrid Plastics (Hattiesburg, MS, USA), and the MTM441 was supplied as prepreg by Solvay Group (Wilton, UK). All samples were used as received without further purification. Moreover, for the development of the ultra-Sthin laminates, a carbon fibre braid provided by A&P Technology Inc. (Cincinnati, OH, USA) was used, as well as unidirectional carbon fibre in tape presentation provided by Oxeon (Borås, Sweden) (Table 1). Kapton^TM^ H manufactured by Dupont was used as a commercial baseline comparison with the laminates.

### 2.2. Layup Configuration of the Thin Films

Both laminates were laid up in a similar fashion, using identical fibre orientations/ply distribution, but with the important distinction that while the MTM44-1 was supplied as a prepreg and could be laid up directly, the cycloaliphatic epoxy system was formed from liquid components and required pre-polymerisation to achieve a low molecular weight, free-standing, integral film.

#### 2.2.1. Layup Procedure for Ultra-Thin Laminates Containing MTM44-1

**Figure 1:**
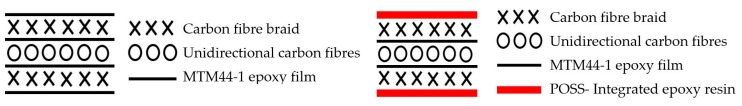
Schematic layup configuration for ultra-thin laminates with MTM44-1 prepreg (left) and with polyhedral oligomeric silsesquioxane (POSS)-integrated CY184 epoxy resin (right).

The method applied to develop ultra-thin laminates was based on the use of a thin metallic plate used to wrap a carbon fibre braid sleeve around to achieve samples of approximate dimensions 80 mm × 100 mm. The carbon fibre braid sleeve was covered by a sheet of resin prepreg and cut into two separate layers so that each layer had a MTM44-1 prepreg sheet on only one side. Several tapes of unidirectional (UD) fibres equal to the same area as that of the carbon fibre braid were attached next to each other. Both sides of this UD layer were stacked with two prepreg resin sheets (Figure 1, left).

All the layers were stacked carefully by peeling the backing paper of the prepreg sheets and placing them in the correct order with the braid fibres being placed perpendicular to the UD fibres.

#### 2.2.2. Layup procedure for ultra-thin laminates containing cycloaliphatic epoxy

The constituents of the resin system (CY 184 (**1**), Aradur 2954 (**2**), EP0409 Glycidyl POSS (**3**)) were mixed to form a liquid resin (see Table 2 for compositions of samples studied).

Samples with different POSS contents (0, 5, 10, 15, and 20 wt%) were considered in this study and the convention (1_x_2_y_3_z_) is used where the subscripts denote the content of each component. Initial attempts were made to achieve a film based on a prepreg system similar to MTM44-1 but, owing to the fluidity of the system, it was proposed to use MTM44-1 to set the internal layup configuration and apply the designed resin system only to the two external layers, as these will be directly in contact with a LEO radiation environment. Therefore, a similar layup configuration was followed as displayed in Figure 1 (right). After, the composite was manufactured, it was left at room temperature to ensure a pre-curing process and, consequently, a partial polymerisation. This process allowed the creation of a stable layup structure where the liquid resin system could be spread using the doctor blade without deforming the carbon fibre braids, leading to a composite configuration as displayed in Figure 1 (right) [22].

### 2.3. Curing Procedure for the Ultra-Thin Laminates

**Figure 2:**
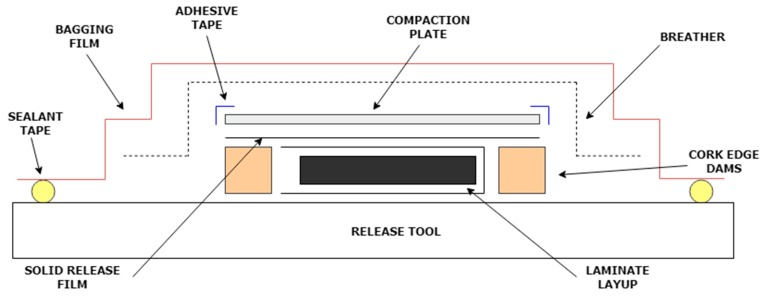
Schematic showing out-of-autoclave (OOA) tooling for ultra-thin laminates.

The baseline MTM44-1 resin system was processed using an out-of-autoclave (OOA) moulding procedure by curing the samples inside an oven with a low-pressure vacuum bag [24]. Being able to manufacture the laminates using this process is an extremely attractive prospect, due to the growing demand for OOA composite manufacturing processes, as they offer a more environmentally sustainable alternative, cost savings, and shorter production times relative to the classic autoclave-based processes [25]. Processing the samples using solely an oven vacuum bag as opposed to using an autoclave may, however, negatively affect fibre volume fraction and void content [26], but as all the samples were manufactured following the same procedure, this process would still allow for the effects of increasing the POSS concentration on laminate samples of similar characteristics to be analysed. The samples were prepared for the oven cure process following the MTM44-1 bagging guidelines [27] with an additional solid release film layer wrapping each sample to prevent any uncontrolled liquid resin from damaging the release tool used (Figure 2). The layup was placed within a vacuum bag with a pressure of 27.5" Hg (The maximum vacuum, which could be achieved after completing the bagging process, was 27.5” Hg, although the MTM44-1 data sheet states that the ideal minimum value is equal to 29” Hg.) which was maintained throughout the oven cure process by means of a vacuum pump. The samples were cured inside an oven for two hours at 130 °C followed by an additional two hours at 180 °C (The first step cure temperature for the CY 184 resin involves a 90 °C dwell step, while a dwell of 130 °C is recommended for the MTM44-1 pre-peg. Consequently, as both samples were processed together, to maintain a consistent thermal history, the slightly higher dwell temperature was used. As a consequence, gelation might have occurred within sections of the resin system, thus impeding the resin from flowing as easily for a uniform distribution along the laminates.) with temperature increment ramps of +2 °C/min. Once the cure cycle had been completed, the tool was allowed to cool to 40 °C prior to retrieving the samples.

### 2.4. Differential Scanning Calorimetry (DSC)

Differential scanning calorimetry (DSC) experiments were performed using a TA DSC Q2000. Hermetically sealed Tzero aluminium pans were used, with sample masses of 9.1 ± 0.2 mg. Samples were equilibrated at 30 °C and then heated to 300 °C at a heating rate of 10 °C/min, unless otherwise stated, with the sample cell kept under a constant nitrogen flow of 50 cm^3^/min. The glass transition temperatures were determined by calculating the midpoint of the curve inflexion during cooling and rescan experiments.

### 2.5. Fourier Transform Infrared (FTIR) Spectroscopy

Surface spectroscopy was measured using Fourier-transform infrared (FTIR) spectroscopy in the Attenuated Total Reflectance (ATR) mode on a Perkin Elmer Fourier-transform infrared (FTIR) spectrometer. A total of 15 scans were performed per sample over a spectral range of 650–4000 cm^−1^ at room temperature and co-added to produce the final spectrum.

### 2.6. Measurements of Surface Roughness and Morphology

Surface topography of the cured polymer and laminate samples was characterised using an Alicona Infinite Focus instrument and Automation Manager surface analysis and imaging software. Surface roughness distribution data and 3D images of the sample surfaces were obtained for scanned areas of 2 mm × 2 mm using a 5× objective. The average surface roughness was calculated from a minimum of 1640 measurement points.

### 2.7. Atomic Oxygen Exposure

The AO exposure test was carried out using a JLS Designs Plasmatherm 550–570, radio frequency plasma generator (located in the Department of Physics, University of Bristol). Four samples from each specimen were placed within the chamber, on an aluminium panel and the following parameters were applied: frequency of 150 W, with a constant pressure of 100 Pa and a constant O_2_ flow of 0.3 NL/min to simulate space conditions. Each sample from each batch was weighed before exposure and then after increments of exposure to AO, the mass losses were determined.

AO fluence was calculated based on the mass loss of a reference Kapton^TM^ H polyimide sample using Equation (1) [28]:

(1) $$F=\frac{∆M_{k}}{\rho_{k}\cdot A_{k}\cdot E_{k}}$$

where *F* is the AO fluence (atom/cm^2^), Δ*M_k_* is the mass loss of Kapton^TM^ H film (g), *ρ_k_* is the density of Kapton^TM^ H (g/cm^3^), *A_k_* is the area of Kapton^TM^ H film (cm^2^), and *E_k_* is the erosion yield of Kapton^TM^ H (cm^3^/atom), which is a constant (3 × 10^24^ cm^3^/atom) measured in the LEO environment [29]. Once the AO fluence was calculated, the erosion yield of tested samples can be calculated using Equation (2):

(2) $$E_{y}=\frac{∆M}{\rho \cdot A\cdot F}$$

where *E_y_* is the erosion yield of tested sample (cm^3^/atom), Δ*M* is the mass loss of tested sample (g), *ρ* is the density of tested sample (g/cm^3^), *A* is the sample surface area (cm^2^) and F is the AO fluence (atom/cm^2^) calculated using Equation (1).

The samples were assigned to six different batches, one sample of each specimen (a total of eight specimens) and exposed to different time intervals (each equivalent to four months in orbit) to analyse a total of 12 months of exposure in space conditions. The cumulative AO fluence received in each exposure run and its equivalent duration in orbit is shown in Table 3. The equivalent duration in orbit of each exposure was calculated based on the data from a mission in the International Space Station launched on June 2001, when the sun entered a period of high activity. During this period the AO fluence in ISS was also maintained at a high level, the total AO fluence of this mission was 3.28∙10^21^ atom/cm^2^ for one year of exposure [28].

### 2.8. Three-Point Bend Test

Each sample was cut into two or three specimens (52 mm × 12.5 mm) both in the direction of the UD plies and perpendicular to them. Three-point bend tests were conducted using a Shimazu mechanical test machine with a 1 kN load cell, following ASTM D7264 standard [30]. Zweben et al. [12] reported that for thin samples, the span to thickness ratio should be more than 60:1 to obtain an accurate result, and consequently a 36 mm span (80:1) was used. The main output of these tests is the effective flexural modulus [31]:

*E* = *PL*^3^/*4bt*^3^*δ*, (3)

where *E* is the effective flexural modulus (GPa), *P* is the force (N), *L* is the support span (mm), δ is the deflection of the centreline of the specimen at the middle of the support span (mm), *b* is the width of specimen tested (mm), and *t* is the thickness of beam tested (mm).

However, the magnitude of flexural modulus is affected by the thickness of the laminate sample and, as this parameter displayed some variation (0.46–0.55 mm), the flexural rigidity [32] was used to characterize the flexural properties of each laminate:

*EI* = *PL*^3^/*48δ* (4)

where *EI* is the flexural rigidity (Nmm^2^), *P* is the force (N), *L* is the support span (mm), and *δ* is the deflection of the centreline of the specimen at the middle of the support span (mm).

## 3. Results and Discussion

### 3.1. Preparation of Self-Sustaining Epoxy Films

Unlike the commercial MTM44-1 system, the cycloaliphatic epoxy (**1**)/amine (**2**) blend was not available as a prepreg and an alternative means of introducing the matrix into the lay-up needed to be found. Initial experiments involved mixing the reactants thoroughly by means of an ultrasonic bath followed by spreading the resultant resin system blend over a solid surface coated with a Frekote release agent using a doctor blade tool to ensure the desired constant thickness of 100 µm. The resultant thin resin film would then be left to dry before it could be retrieved as a self-sustaining film, which could be peeled from the solid surface and transferred directly to the laminate layup. The free-standing film was found to have a cured T_g_ of 71 °C using DSC.

### 3.2. Preparation of Ultra-Thin Laminates and Spectroscopic Characterisation of the Resin Blends

**Figure 3:**
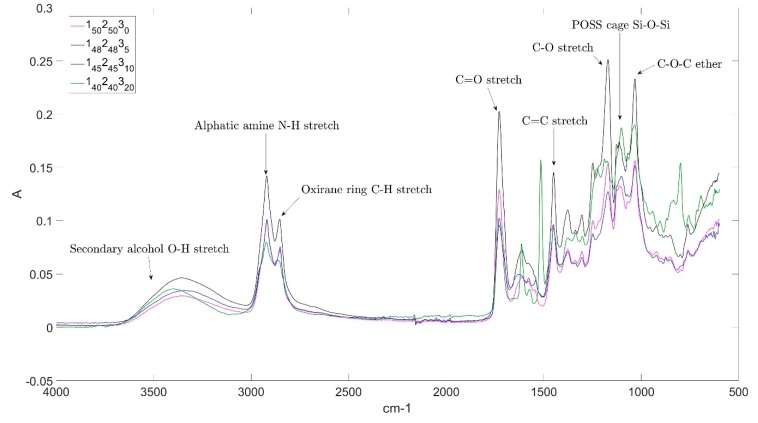
Infrared spectra of cured epoxy resin blends as a function of POSS content.

The ultra-thin laminates produced using the compaction method were not completely flat (showing a typical deviation of ±0.04 mm at the midpoint). It was noted that the compaction plate used to apply pressure to the laminate samples during the oven vacuum bag cure did not completely cover them. This effect may have caused a differential in the force to which the samples were subjected and, combined with any spring-back behaviour associated with other residual stresses arising from the cure process, may have resulted in the slight curvature observed following cure. FTIR spectral analysis of the epoxy blends was performed to confirm the incorporation of the POSS reagent (**3**) and the spectra are presented as a function of POSS content (Figure 3). Selected characteristic bands are given in Table 4, along with proposed spectral assignments. The blends containing POSS all display the key spectral characteristic of an intense band around 1100 cm^−1^, which corresponds to the asymmetrical stretching vibration from the Si-O-Si bond in the silsesquioxane cages [33]. As the POSS concentration is increased, an apparent increase in the O-H stretching band is observed. This is an indication of the opening of the epoxy rings when reacting with the POSS and diamine molecules, leading to the formation of hydroxyl groups (and is thus a measure of the degree of polymerisation).

### 3.3. Measurements of Surface Topography of the Laminates

**Figure 4:**
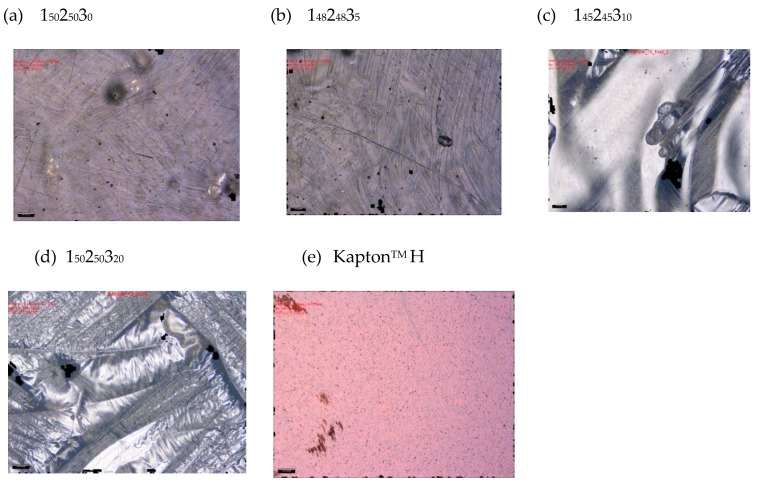
Microscopies of top (exposed) surfaces of all cured samples and Kapton^TM^ H before exposure (N.B. scale bar represents 200 μm).

Minimising the surface roughness of the laminates is a key step towards optimising their performance, as the probability of a damaging reaction with energetic photons and AO present in the LEO environment increases with the surface area of the exposed material [10]. Additionally, the presence of voids and a high surface roughness is particularly severe for ultra-thin laminates such as those reported here, as any surface imperfection has a much larger effect on their thickness, leading to a deterioration of their mechanical properties. A visual inspection of all the samples confirmed that all but the bottom surface of the pure epoxy sample (i.e., containing 0 wt% POSS) contained surface imperfections, including voids and resin trails, especially in the centres of the samples (Figure 4). The top surface of the pure epoxy (1_50_2_50_3_0_) and both surfaces of the sample containing 5 wt% POSS (1_43_2_43_3_5_) were slightly smoother than the remaining samples. When the four samples were being manufactured in batches, film samples were stored in a fridge to arrest polymerisation. However, it is known [34] that even freezing cannot entirely stop the autocatalytic epoxy cure.

This might explain the slight differences in cure state and why one side of the 0% POSS samples appeared to have a significantly flatter surface with fewer imperfections and the 5% POSS resin had smoother surfaces than the other two POSS-integrated samples. Laminates incorporating resins with a higher viscosity are also more susceptible to the formation of voids and resin accumulations on the surface as it is more difficult for the resin to penetrate the carbon fibre layers and fill any voids below the surface [18]. Glycidyl POSS is a relatively viscous liquid and the epoxy blends samples with higher POSS concentrations were significantly more viscous and more susceptible to the formation of surface defects. Nevertheless, as only one sample was manufactured for each POSS concentration, it was not possible to confirm whether the higher degree of polymerisation of the epoxy resins was the reason behind the high surface roughness of some samples or whether the POSS nanoparticles had an influence on this property. As the centre of the laminates displayed a greater number of surface imperfections, this may have been due to an uneven heat distribution during the cure process (i.e., if the sides of the samples were exposed to more heat originating from the oven, then the liquid resin system in these regions would had been able to flow more readily, thus leading to a more uniform distribution of the resin). As most of the manufacturing processes carried out throughout this study were of an iterative nature with over a hundred epoxy resin films being fabricated over the course of the project, a decision was made to simply use of a water bath for 5 minutes to mix the POSS additive inside the resin system (Suliga [22] had previously determined that octafunctional POSS disperses well within the cylcloaliphatic epoxy resin under these conditions).

**Figure 5:**
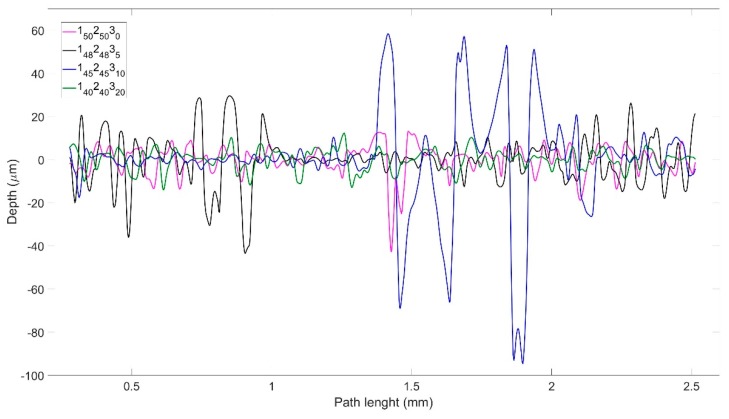
Topographical analysis of surface roughness of cured laminates with different POSS content.

The bottom surface of the laminate sample containing 0% POSS displayed a significantly smoother surface compared to those of the nanocomposites (Figure 5). Additionally, there appears to be no periodicity to the features in the distribution of the surface roughness data along the samples’ lengths. Consequently, it may be concluded that this parameter does not depend on the distribution of the carbon fibre braids which contain a repeating pattern, thus indicating that the surface roughness is more dependent on resin accumulations than voids forming on the carbon fibre braid structure.

The surface roughness distribution of the scanned surfaces also exhibits significantly different amplitudes for the different samples, e.g., as the two surfaces of the 0% POSS sample have the same amount of POSS and the resin systems of the other two surfaces were spread over the carbon fibres at the same time, these data therefore appear to indicate that the two theories which were previously discussed for the dissimilarities in the amount of surface imperfections combined could justify the differences in the surface uniformity of the samples. While determining the surface topography of the samples, it was found that the depth of the surface imperfections for the blends containing 10 wt% and 20 wt% POSS was too large for the Alicona instrument to obtain an accurate representation of the surfaces, with these being represented as gaps in the surface by the program. Consequently, it was only possible to obtain reliable data for the surface roughness of the epoxy samples containing 0 wt% and 5 wt% POSS.

### 3.4. Three-Point Bending Analysis of Virgin Cured Laminates

**Figure 6:**
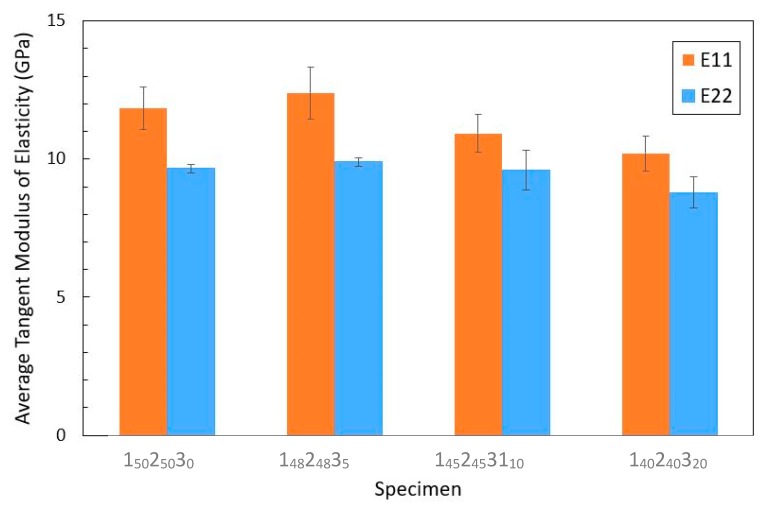
Graphs displaying the variation in the E_11_ and E_22_ flexural moduli of the samples as a function of POSS content.

The tangent modulus data obtained for the virgin, cured ultra-thin laminates using 3-point bend tests are shown in Figure 6. Each bar represents the average tangent modulus of elasticity calculated in either the E_11_ direction, parallel to the UD fibres or the E_22_ direction, perpendicular to them.

The data used to obtain the flexural modulus of each sample are displayed in Table 5. Error bars are shown that represent one standard deviation between the tangent modulus values of the specimens used to calculate each average. As previously discussed, the samples were not perfectly planar and this mild curvature could potentially affect the mechanical testing results, as the calculations involved assume an initial flat surface. With curved specimens, a torque may be generated as the specimens are contacted by the loading pin, thus leading to an underestimation of their flexural properties. To avoid the effects of the thickness of the laminate sample, the flexural rigidity was used to characterize the flexural properties of each laminate.

Initially, the stiffest of the matrices is the unmodified base epoxy and, at low concentrations of 5%, the addition of the POSS nanofiller results in an increase in the flexural modulus of the specimens followed by a decrease in this property as the POSS concentration is further increased; this is true for both the E_11_ and E_22_ values. The POSS cages have a higher modulus than the polymer chains and these rigid structures can form bonds with the organic molecular chains of the epoxy resin matrix and restrict their movement, thus improving their flexural properties [35]. It is important, however, to note that the magnitude of the increase in the average tangent modulus of the samples is within the expected error associated with the test. Consequently, in order to verify this theory, further work should be conducted with larger sample sizes so that the uncertainty from the values obtained may be reduced. Once the nanoparticle concentrations reach a certain limit, the mechanical properties of the specimens start to drop considerably. Owing to the high surface energy of these nanoparticles, once the filler concentration becomes too high, it is extremely difficult to ensure its uniform dispersion inside the resin and the particles start to agglomerate, which results in the formation of local stress concentrations. This leads, in turn, to a drop in the Young’s Modulus of the laminates and an increase in their susceptibility to fracture [36]. Consequently, for this set of manufactured samples there is a limiting POSS concentration of up to 10 wt% before the nanoparticle agglomerations lead to deterioration in the mechanical properties.

For all the samples, the average tangent modulus is significantly larger along the E_11_ axis, which is aligned with the UD fibres, indicating that the flexural properties of the laminates are more dependent on the orientation of the UD fibres than that of the braid fibres. A study presented by Swanson et al. [37] found that braid fibres tend to show a reduction in their mechanical properties compared with UD fibres, due to the undulation in the braid fibre paths which results in the formation of stress concentrations. Owing to the relatively small size of the sample, in order to be able to retrieve a larger number of specimens, the length of the specimens extending over the support pins was relatively short compared to the support span. When the specimens were subjected to high loads, it could be observed that some slippage was occurring between the specimens and the supporting pins. The results obtained at high deflections were therefore deemed to be inaccurate and the specimens were not tested up until the fracture point; consequently, no data on the flexural strength of the samples could be collected. It is also important to note that the maximum recommended span-to-depth ratio for the specimens according to the ASTM standards is 60:1, which is significantly smaller than the 80:1 value used during the tests. Owing to the extremely thin nature of the specimens used, it was not possible to select a smaller ratio as the fixtures of the Shimadzu machine used were constrained to a minimum distance. This results in an underestimation of the modulus of the specimens, as the small deflections assumption from the elementary bending theory equations is no longer valid.

### 3.5. Characterisation of Ultra-Thin Laminates after Exposure to AO

#### 3.5.1. Mass loss as a Result of AO Exposure

Each sample was weighed before and after every exposure to keep track of any mass changes during exposure and these data are shown in Table 6.

A polyimide witness sample (Kapton^TM^ H) was included to provide a means of calibrating the response, since the behaviour of this material is well documented, and this was used to calculate the AO fluence. The data show that the base amine-cured cycloaliphatic epoxy, containing no POSS, (1_50_2_50_3_0_) lost 4.1 % of its mass over the same 12-months of simulated exposure, while increasing amounts of POSS reduced the amounts of mass lost, with the blend containing 20 wt% POSS displaying the lowest mass loss (2.5 %) after the equivalent of 12 months of AO exposure.

Two kinds of AO are involved in the degradation mechanisms: the ground state (3P) and electronically excited (1D) form [38]. The ground state form, O (3P), can abstract a hydrogen atom in the polymer structure to form a hydroxyl group with activation energies of 28.9 kJ/mol. (where the hydrogen atom is primary), 18.8 kJ/mol. (secondary), and 13.8 kJ/mol. (tertiary). The excited form, O (1D), can also react with more complex aromatic compounds by abstraction of a hydrogen atom from the phenyl ring to form a hydroxyl radical and a phenyl radical, or addition of AO to form various possible products, which either undergo rearrangement to a more stable phenolic structure or participate in condensation and polymerization reactions. However, the excited form, O (1D) is more likely to undergo insertion into the C-H bond of an alkyl substituent with no energy barrier, e.g., leading to hydroxylated species. The incorporation of POSS into the epoxy introduces species containing Si-O bonds, which are significantly stronger than their carbon analogue e.g., bond energies of selected groups: C-C 370 kJ/mol., C-H 435 kJ/mol., C-O 360 kJ/mol., C-N 305 kJ/mol., Si-O 460 kJ/mol., Si-C 318 kJ/mol.) [39]. This increased dissociation energy improves both thermal and thermo-oxidative stability of the resulting nanocomposites, and furthermore, the POSS reagents are transparent to UV radiation. During AO exposure, the POSS moieties undergo cage opening and recombination with the oxygen to develop a protective, silica-based coating on the surface of the laminates.

Determination of the erosion yields (E_y_) was also carried out, but the trends are hard to discern (the sample sizes and consequent mass losses are small), but for completeness the data are supplied in the Supplementary Materials, Figure S2. The unmodified blend (1_50_2_50_3_0_) appears to display an erosion yield (E_y_) that fluctuates (presumably due to the small masses involved) around E_y_ = 4 × 10^-24^ cm^3^/atom over the equivalent of 12 months of LEO exposure. This compares well with E_y_ = 3.9 × 10^-24^ cm^3^/atom [40] for MTM44-1 laminates, previously reported by our team. The former figure was recorded using the same apparatus for AO fluence up to 15 × 10^20^ atom/cm^2^, while the latter figure was recorded using a stronger plasma/VUV source. Similarly, Banks et al. [41] reported an erosion yield of 4.2 × 10^-24^ cm^3^/atom for an aromatic epoxy EP. The blends containing POSS all initially showed similar behaviour: the erosion yields were initially high and proportionate to the POSS content, but the E_y_ values appear to rise again, and this is particularly marked in the case of the 1_48_2_48_3_5_ blend, perhaps indicating further damage to the surface. More detailed work is necessary to explore this.

#### 3.5.2. Three-Point Bending Analysis of Cured Laminates Following Exposure to AO

**Figure 7:**
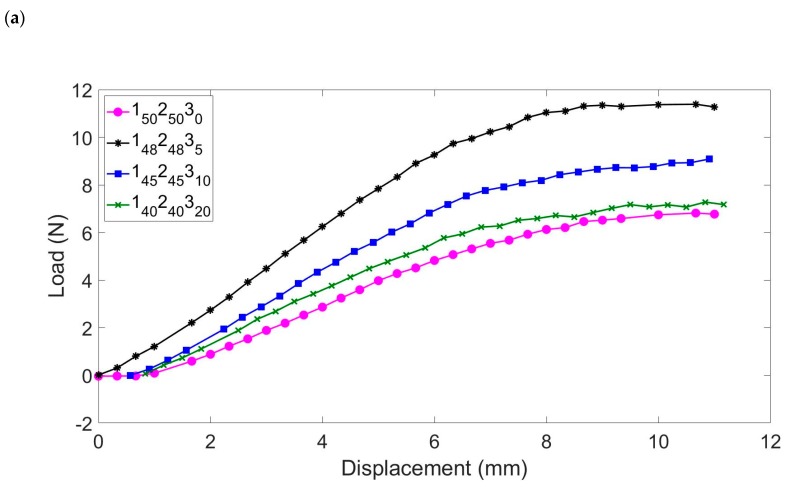

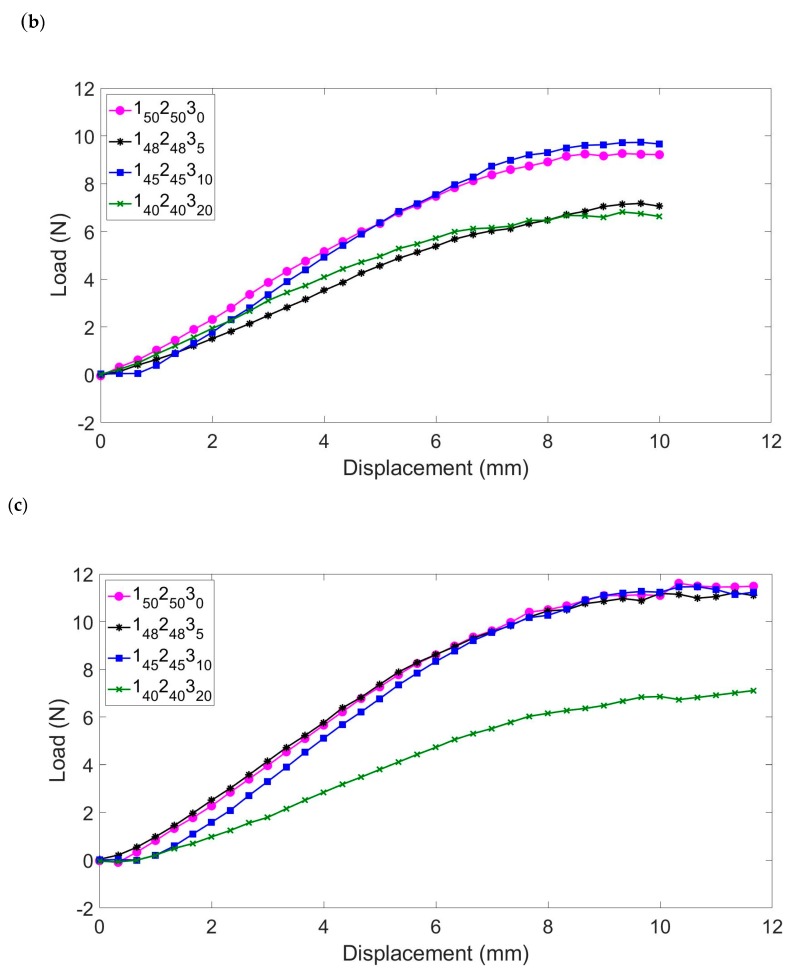
Three-point bending test applied to cured samples after the equivalent of (**a**) 4, (**b**) 8, and (**c**) 12 months in a simulated LEO environment.

The result of AO exposure on the mechanical (flexural) properties of the laminates is shown in Figure 7. After a simulated exposure of 4 months in LEO, the POSS-epoxy nanocomposites offer greater stiffness than the unmodified base epoxy, 1_50_2_50_3_0_, and the stiffest of the matrices contains the lowest POSS content (i.e., the stiffness falls as the POSS content increases). According to data acquired in-orbit during NASA’s Materials International Space Station Experiments (MISSE) programme [28], the resin matrix is significantly more susceptible to the effects of AO than the reinforcing fibres: the erosion yield of a generic epoxy resin is about 10 times greater than that recorded for pyrolytic graphite (a model for the carbon fibres). Thus, after the majority of the surface resin has been eroded, the exposed carbon fibre is relatively resistant to erosion, although not entirely inert. Therefore, after the first exposure, the flexural properties will not change significantly unless the fibres become seriously eroded and damaged, which requires a higher AO fluence than was achieved in this work.

However, as the AO fluence grows, then the stiffness of the laminates varies, so that after a 12-month period of simulated exposure, the laminates display very similar behaviour (with the exception of 1_50_2_50_3_0_), becoming significantly stiffer as a result of crosslinking and AO reaction to form a silica surface layer. Having assessed the bulk properties of the laminates, more detailed analysis was performed on the surfaces of the laminates to confirm the mechanism responsible for the increases in flexural properties.

The three-point bend tests were conducted on a total of 58 laminate test specimens following AO exposure and the data are presented in Table 7. The main output of these tests is the effective flexural modulus; however, achieving a consistent laminate thickness in ultra-thin laminates is difficult, which leads to some variation in the calculated flexural modulus. Consequently, to avoid the effects of the thickness of the laminate sample, the flexural rigidity was used to characterize the flexural properties of each laminate.

#### 3.5.3. Measurements of Surface Topography of the Laminates after Exposure

**Figure 8:**
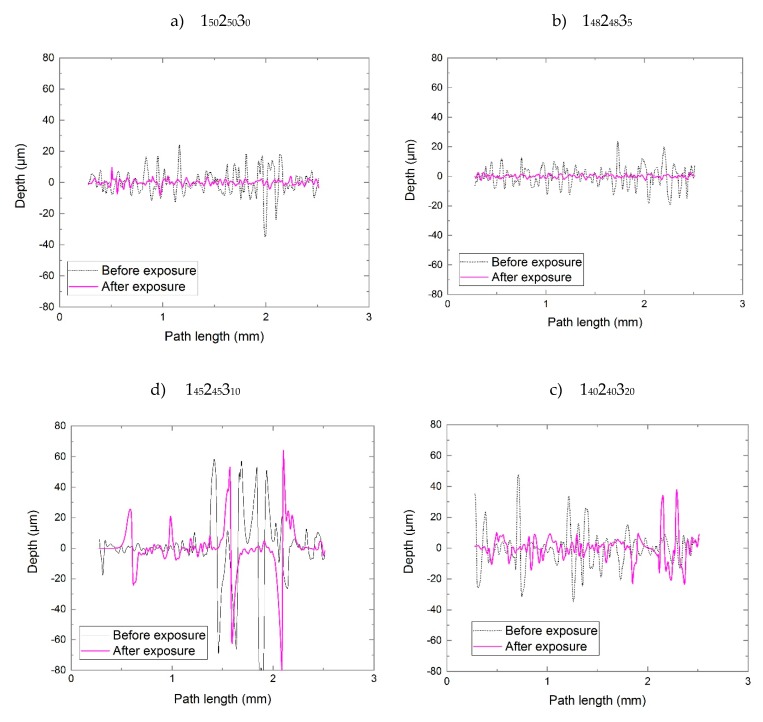
Roughness topographic analysis of virgin cured laminates as a function of POSS content: (**a**) 0%, (**b**) 5%, (**c**) 10%, (**d**) 20% and after 12 months of exposure in simulated LEO.

A baseline comparison was made using the commercial material, MTM44-1, which contains no POSS, and which we have explored in detail in a previous publication [40]. After a relatively short period of simulated LEO exposure (equivalent to less than 3 months), the reinforcement fibres are observed clearly using the Alicona Microscope (Supplementary Materials, Table S1), indicating the almost complete loss of the matrix resin from the surface. This agrees well with our previous studies with this material. A similar finding is obtained for the unmodified cycloaliphatic epoxy, 1_50_2_50_3_0_, for which reinforcement fibres are also visible by 8 months, although the increased stability towards VUV radiation appears to impede this process. As the surface topography of the samples was being obtained (see Supplementary Materials, Table S2 for MTM44-1 data), the non-uniformity of the surfaces of the laminates containing 10 wt% and 20 wt% POSS (1_45_2_45_3_10_ and 1_40_2_40_3_20_) proved challenging for the Alicona microscope, leading to the display of small gaps in the surface data (Supplementary Materials, Table S3). After the exposure, it was observed that the silicon-rich layer became less deep, but presumably both the silicon layer and the exposed cycloaliphatic epoxy below should allow the laminate to resist VUV radiation for a longer period than the corresponding aromatic epoxy (MTM44-1). The laminates containing 10 wt% and 20 wt% POSS show a very different behaviour and, as expected, the reaction of the AO with the POSS integrated into the blends leads to the formation of a translucent top layer, which shields the matrix from further degradation. The formation of a smooth, protective layer is also confirmed by the measurements of surface roughness (Figure 8), where the irregular surfaces of 1_45_2_45_3_10_ and 1_40_2_40_3_20_ are particularly evident (see also Supplementary Materials, Table S4).

#### 3.5.4. Spectroscopic Characterisation of the Laminates after Exposure

**Figure 9:**
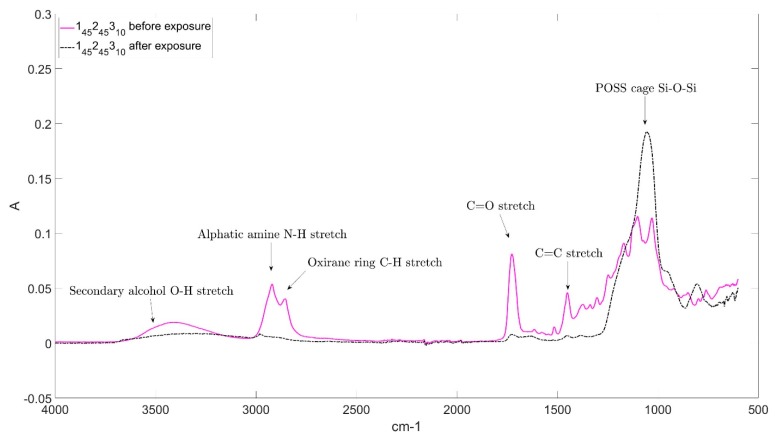
Infrared spectra of cured 1_45_2_45_3_10_ laminates before and after 12 months of exposure in simulated LEO.

FTIR spectra are shown for the cured 1_50_2_50_3_0_ sample before and after the conclusion of the LEO exposure experiment (Figure 9). The surface of the exposed sample is dominated by signals characteristic of the POSS cage and silica (see Supplementary Materials, Table S5).

## 4. Conclusions

Four CFRP samples were manufactured with increasing amounts of the POSS nanoparticles at 0%, 5%, 10%, and 20% of the total resin system mass. A series of tests were conducted to evaluate the validity of the manufacturing process and the effect of increasing the POSS concentration inside the cycloaliphatic epoxy resin on the properties of ultra-thin laminates. Surface scanning reveals that the unmodified epoxy sample (containing no POSS) has a more uniform surface than the other samples, with more granular surfaces observed for the nanocomposites. The flexural properties of the laminates were found to improve slightly from 0% to 5% POSS concentration before decreasing steadily after agglomerations of the additive are formed. After the laminates were exposed to AO, the formation of a protective layer was observed, especially in those containing 10 wt% and 20 wt% POSS. Microscopic and topographical analysis reveals an increasingly uniform protective layer following exposure. The presence of POSS reduces the erosion yield, compared with the unmodified cycloaliphatic epoxy, with the erosion yield falling proportionately as the amount of silicon is increased. The samples containing POSS had superior flexibility properties when compared to laminates containing the commercial epoxy MTM44-1; the highest flexural modulus and strength was recorded for the laminate containing 5 wt% POSS. The challenge is to achieve a suitable manufacturing method capable of incorporating sufficient POSS content (some 5–10 wt% in this case) without compromising the processing characteristics or the final mechanical properties. In this case, the laminates were produced on a relatively small scale, so lab-based methods were employed to obtain the films, whereas the logical step would be to concentrate on optimising the preparation of the films to produce homogeneous dispersions of POSS; this aspect is now receiving our attention.

## Figures and Tables

**Table 1:** The structures of the chemicals used in this work.

Element	Trade Name	Structure
**1**	CY 184	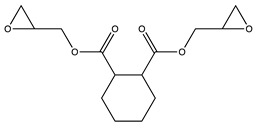
**2**	Aradur 2954	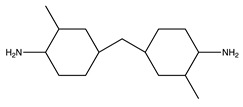
**3**	EP0409 glycidyl POSS	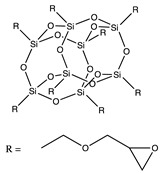
**4**	MTM44-1	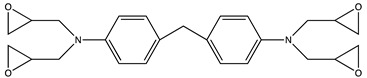
		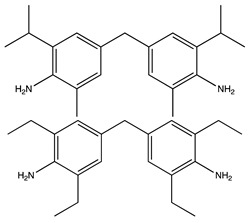

**Table 2:** The compositions of the chemical blends used in this work.

Sample	Composition (grammes) of Components		
	CY184 (1)	Aradur 2954 (2)	POSS (3)
Kapton^TM^ H (film)			
MTM44-1			
1_50_2_50_3_0_	1.5	1.5	0
1_48_2_48_3_5_	1.44	1.44	0.12
1_45_2_45_3_10_	1.35	1.35	0.3
1_40_2_40_3_20_	1.2	1.2	0.6

**Table 3:** Batches of samples tested under atomic oxygen (AO) fluence for each exposure.

Batch	Exposure Time (mins)	Total AO Fluence (×10^20^ atom/cm^2^)	Equivalent Duration in Orbit (Months)
1	30	3.59	2
2	60	5.39	4
3	90	5.75	6
4	120	6.47	8
5	150	7.92	10
6	180	9.34	12

**Table 4:** Characteristic FTIR (Fourier-transform infrared spectroscopy) of the absorbance bands for the cured 1_x_2_y_3_z_ samples (containing POSS).

Wavenumber (cm^−1^)	Intensity	Functional Group
850	Weak	POSS cage, Si-C stretch
910	Weak	Oxirane ring, C-O asymmetric stretch
1035	Medium, Sharp	C-O-C ether symmetric stretch
1100	Medium	POSS Cage Si-O-Si, asymmetric stretch
1170	Medium, Sharp	Oxirane ring, C-O stretch
1450	Medium, Sharp	CH_3_ and CH_2_, C-H deformation
1450	Medium, Sharp	Aromatic ring, C=C stretch
1520	Weak	Amine, N-H stretch
1725	Strong, Sharp	Saturated carbonyl, C=O stretch
2850	Medium	Oxirane ring, C-H stretch
2920	Medium	Aliphatic amine, N-H stretch
3500	Strong, Broad	Secondary alcohol, O-H stretch

**Table 5:** Dimensions obtained from the 3-point bending test for specimens and the calculated tangent moduli of elasticity.

Sample	Replica	Thickness (mm)	Width (mm)	Linear Elastic Response Gradient	Tangent Modulus Elasticity (GPa)	Orientation	Flexural Rigidity (Nmm^2^)
	10	0.54	14.31	2.52	13.06		
	Mean				12.40 (σ = 0.94)		2211.3 (σ = 336.78)
1_45_2_45_3_10_	12	0.51	13.33	1.33	8.79	E_22_	1292.76
	13	0.49	14.26	1.46	10.18		1419.12
	14	0.51	14.85	1.65	9.80		1603.8
	Mean				9.59 (σ = 0.72)		1438.56 (σ = 156.43)
	15	0.53	13.08	1.75	10.46	E_11_	1701
	16	0.50	12.59	1.54	11.42		1496.88
	Mean				10.94 (σ = 0.68)		1598.94 (σ = 144.33)
1_40_2_40_3_20_	17	0.46	14.05	1.07	9.09	E_22_	1040.04
	18	0.49	12.35	1.12	8.97		1088.64
	19	0.46	12.13	0.82	8.07		797.04
	Mean				8.79 (σ = 0.56)		975.24 (σ = 156.22)
	20	0.47	14.37	1.25	9.75	E_11_	1215
	21	0.47	14.32	1.36	10.64		1321.92
	Mean				10.20 (σ = 0.63)		1268.46 (σ = 75.60)

**Table 6:** Mass loss analysis of samples with different POSS content and different curing procedures for 12 months in simulated low Earth orbit (LEO) conditions.

Specimen Group	POSS Content (%)	Weight before Exposure (g)	Weight after Exposure (g)	Weight Loss (%)
1_50_2_50_3_0_	0	0.465	0.446	4.1
1_48_2_48_3_5_	5	0.452	0.438	3.1
1_45_2_45_3_10_	10	0.535	0.519	2.8
1_40_2_40_3_20_	20	0.436	0.425	2.5
Kapton^TM^H		0.124	0.098	20.9

**Table 7:** Three-point bending data for the cured laminates as a function of POSS content after 4, 8, and 12 months of atomic oxygen exposure.

Specimen	Months of Exposure	Width (mm)	Thickness (mm)	Linear Region Slope (N/mm)	Modulus of Elasticity (GPa)	Flexural Rigidity (Nmm^2^)
1_50_2_50_3_0_	4	12.97	0.53	1.01	6.08	977.60
	8	12.02	0.58	1.72	7.19	1404.99
	12	12.44	0.55	1.72	7.85	1670.97
Mean					7.04 (σ = 0.89)	1351.19 (σ = 349.80)
1_46_2_46_3_5_	4	13.40	0.59	1.74	7.38	1692.67
	8	12.79	0.53	1.02	5.58	990.46
	12	13.13	0.63	1.57	5.60	1531.27
Mean					6.19 (σ = 1.03)	1404.8 (σ = 367.79)
1_45_2_45_3_10_	4	13.71	0.59	1.30	5.40	1266.55
	8	14.16	0.63	1.57	5.19	1530.20
	12	14.83	0.65	1.78	5.09	1727.38
Mean					5.23 (σ = 0.16)	1508.04 (σ = 231.21)
1_40_2_40_3_20_	4	13.86	0.54	1.04	5.56	1011.87
	8	12.34	0.56	1.11	5.96	1077.18
	12	12.57	0.54	0.91	4.83	888.54
Mean					5.45 (σ = 0.57)	992.53 (σ = 95.79)

## Supplementary Materials

The following are available online, Figure S1: Examples of applications of deployable structures, Figure S2: Erosion yield obtained for all the POSS content samples after exposure, Table S1: Microscopy for cured laminate surface with MTMM4-1 content and virgin KaptonTM H following exposure to AO in simulated space conditions for a period of 12 months, Table S2: 3D Topographical analysis for cured laminate surface with MTMM4-1 content and virgin KaptonTM H following exposure to AO in simulated space conditions for a period of 12 months, Table S3: Microscopy for cured laminate surfaces as a function of POSS content following exposure to AO in simulated space conditions for a period of 12 months, Table S4: 3D Topographical analysis for cured laminate surfaces as a function of POSS content following exposure to AO in simulated space conditions for a period of 12 months, Table S5: Characteristic FTIR of the absorbance bands for the cured 145245310 samples before and after 12 months of exposure in simulated LEO.

## Abbreviations

AO	Atomic oxygen
CFRP	Carbon fibre reinforced polymer
DMA	Dynamic mechanical analysis
DSC	Differential scanning calorimetry
E	Erosion Yield
FTIR	Fourier-transform infrared
GFRP	Glass fibre reinforced polymer
LEO	Low Earth orbit
MISSE	Materials International Space Station Experiment
NASA	National Aeronautics and Space Administration
OOA	Out-of-autoclave
POSS	Polyhedral oligomeric silsequioxane
SEM	Scanning electron microscopy
TGDDM	Tetraglycidyldiaminodiphenylmethane
UD	Unidirectional
VUV	Vacuum ultraviolet
wt%	Weight content in percentage
