# Supplementary material for: Development of Cycloaliphatic Epoxy-POSS Nanocomposite Matrices with Enhanced Resistance to Atomic Oxygen
Source: Molecules. 2020 Mar 25;25(7):1483. doi: 10.3390/molecules25071483 (PMC7180924; doi:10.3390/molecules25071483)
Supplement: Supplementary file 1 [file molecules-25-01483-s001.pdf]

molecules

Supplementary Materials

Development of Cycloaliphatic Epoxy-POSS Nanocomposite Matrices with Enhanced Resistance to Atomic Oxygen

Mayra Y. Rivera Lopez [1], Javier Martin Lambas [1,†], Jonathan P. Stacey [1], Sachithya Gamage [1], Agnieszka Suliga [1,‡], Andrew Viquerat [1], Fabrizio Scarpa [1] and Ian Hamerton [1,*]

[1] Bristol Composites Institute (ACCIS), Department of Aerospace Engineering, School of Civil, Aerospace, and Mechanical Engineering, Queen's Building, University of Bristol, University Walk, Bristol, BS8 1TR, United Kingdom

[2] Department of Mechanical Engineering Sciences, Faculty of Engineering and Physical Sciences, University of Surrey, Guildford, Surrey, GU2 7XH, United Kingdom

* Correspondence: ian.hamerton@bristol.ac.uk

† Present address: National Composites Centre, Feynman Way Central, Bristol and Bath Science Park, Emersons Green, Bristol BS16 7FS, United Kingdom

‡ Present address: European Space and Technology Research Centre, European Space Agency, 2201 AZ Noordwijk, Netherlands.

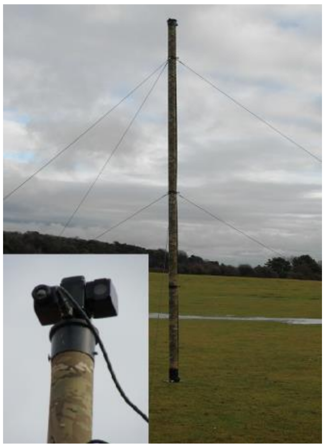

a) RAMM Integrated Antenna Mast System [7]

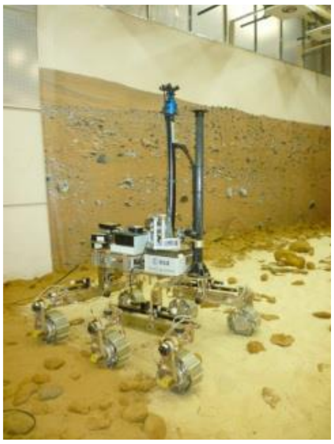

b) Motorised Camera mast on EADS Astrium Mars Rover [7]

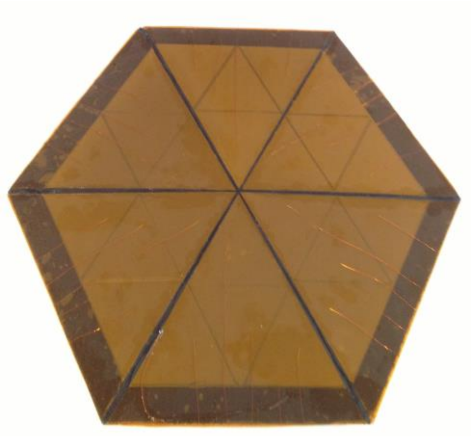

c) Mirror Prototype [8]

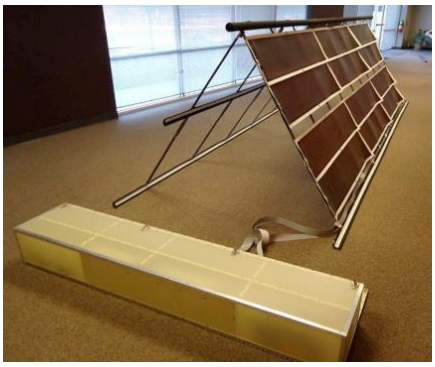

d) Roll Out Solar Power System [7]

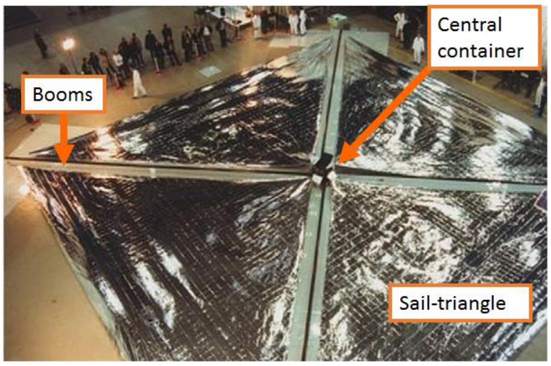

e) Solar Sail components [9]

Figure S1. Examples of applications of deployable structures.

Table S1. Microscopy for cured laminate surface with MTMM4-1 content and virgin Kapton™ H following exposure to AO in simulated space conditions for a period of 12 months.

	MTM44-1	Kapton™ H
0 Months (before exposure)	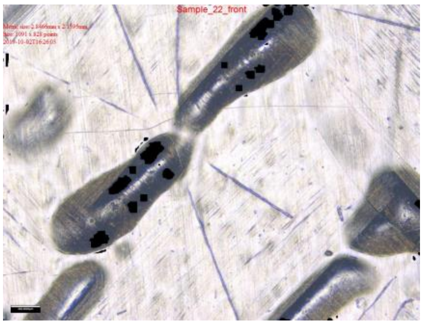	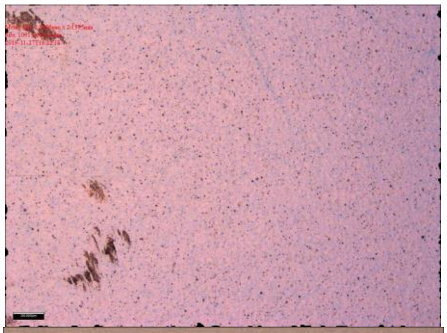
4 months	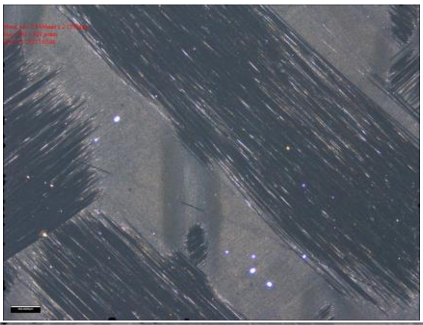	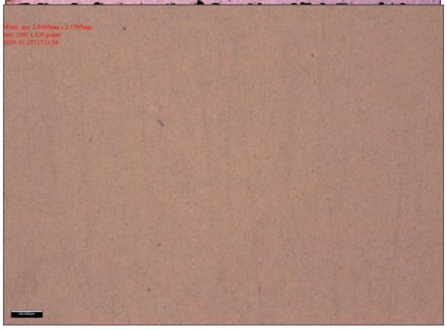
8 Months	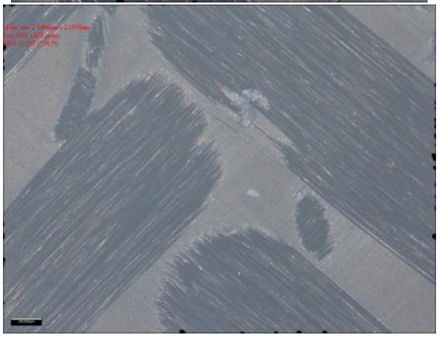	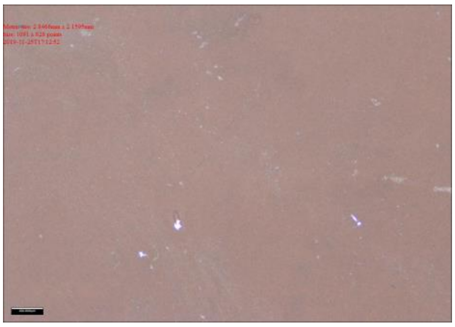
12 Months	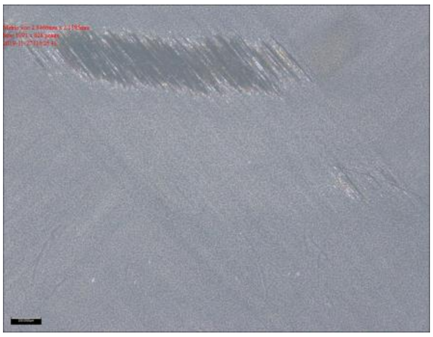	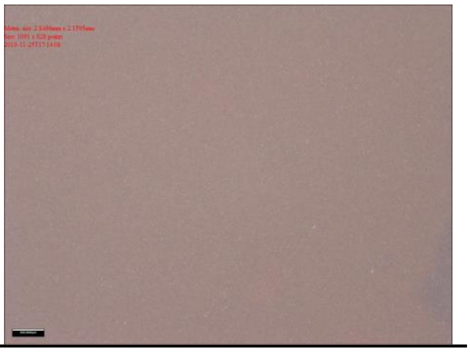

Table S2. 3D Topographical analysis for cured laminate surface with MTMM4-1 content and virgin Kapton™ H following exposure to AO in simulated space conditions for a period of 12 months.

	MTMM4-1	Kapton™ H
0 Months<br>(before exposure)	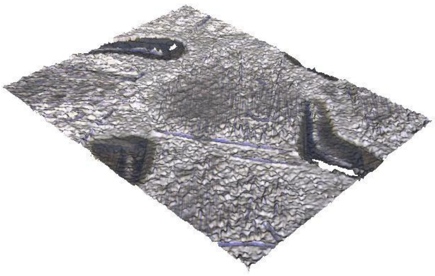	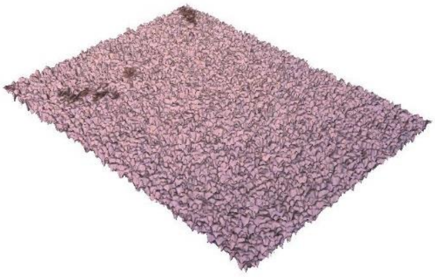
4 months	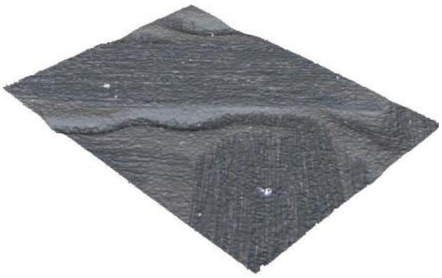	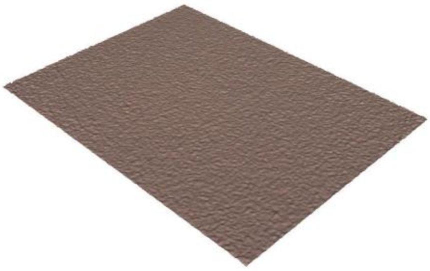
8 Months	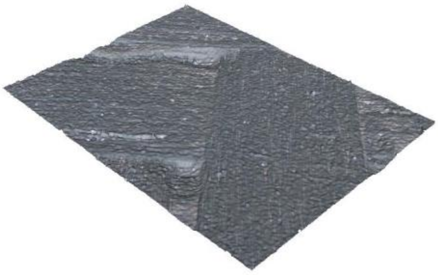	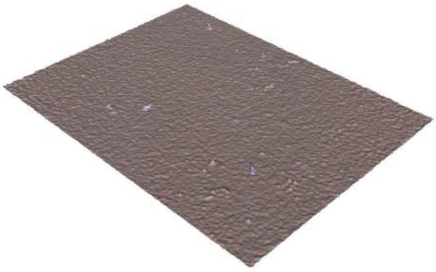
12 Months	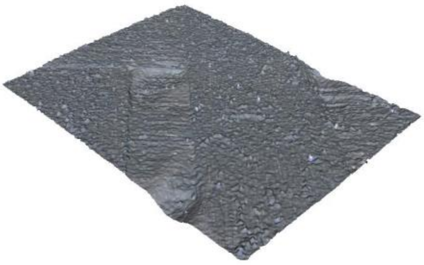	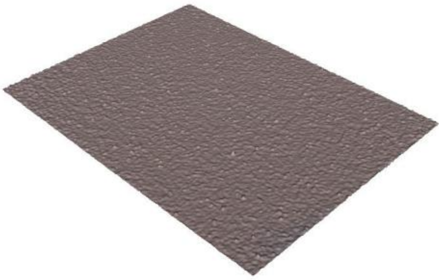

Table S3. Microscopy for cured laminate surfaces as a function of POSS content following exposure to AO in simulated space conditions for a period of 12 months.

Time of AO exposure	$1_{50}2_{50}3_{0}$	$1_{48}2_{48}3_{5}$
0 Months (before exposure)	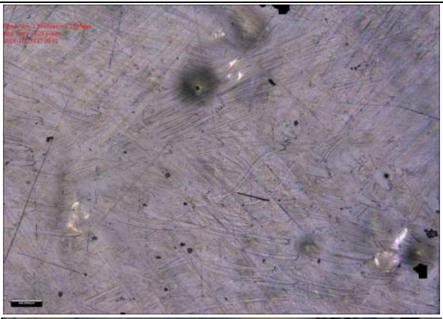	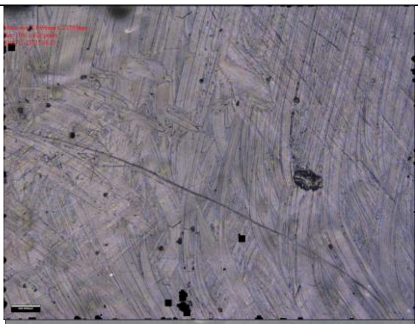
4 months	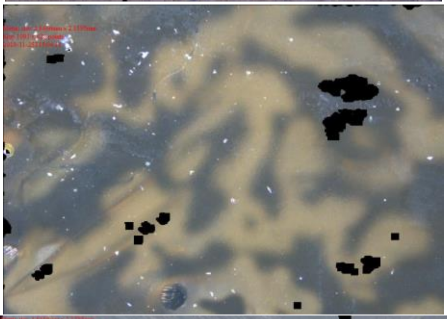	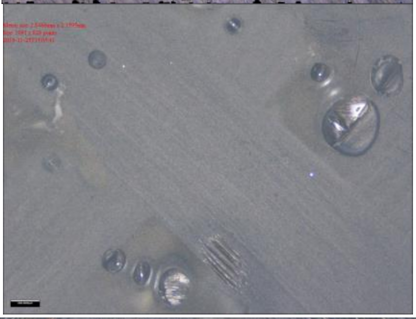
8 Months	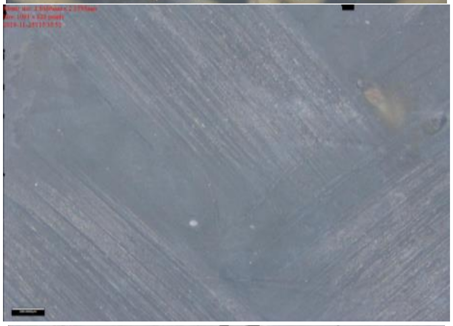	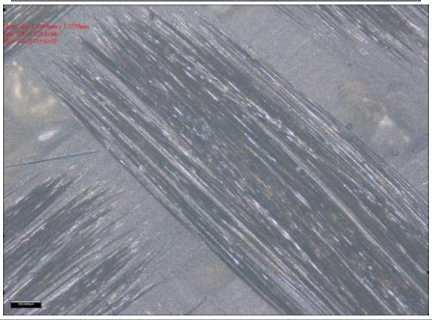
12 Months	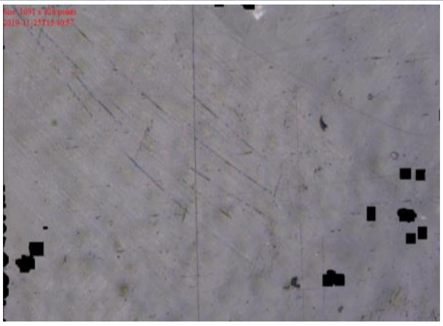	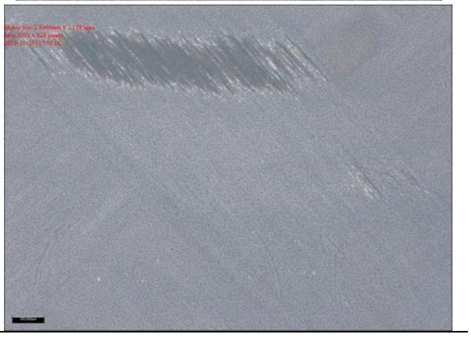
	$1_{45}2_{45}3_{10}$	$1_{40}2_{40}3_{20}$
0 Months (before exposure)	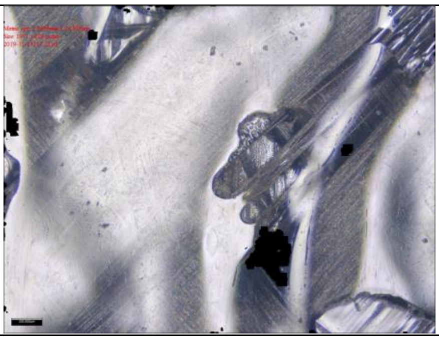	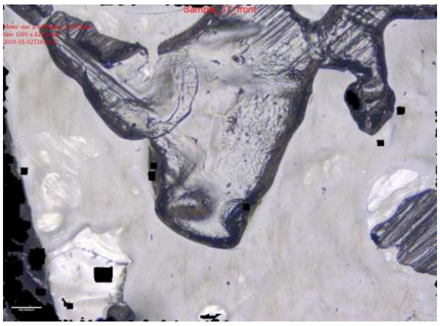

4 months	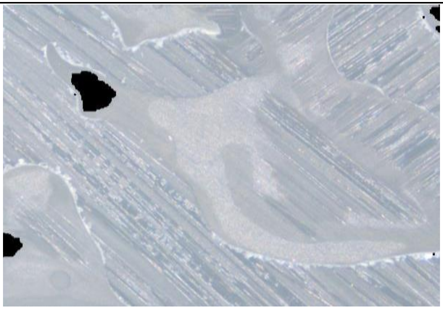	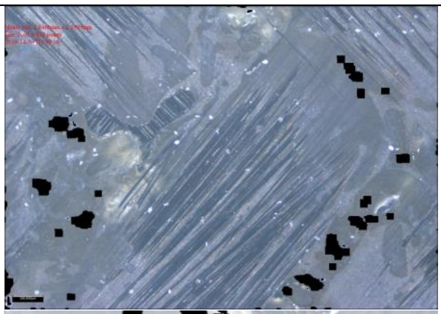
8 Months	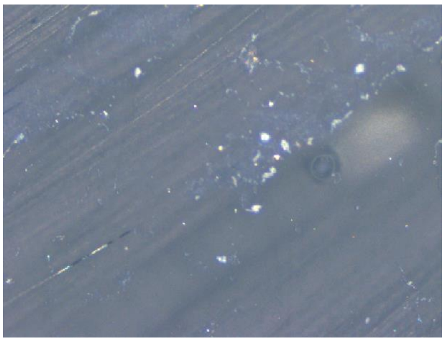	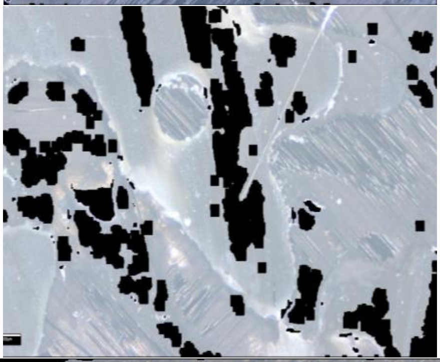
12 Months	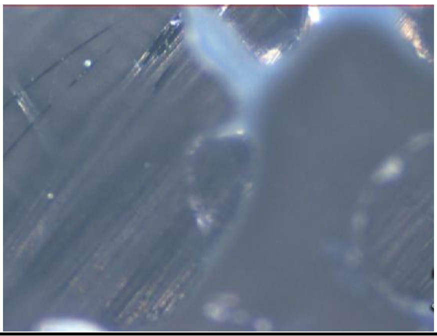	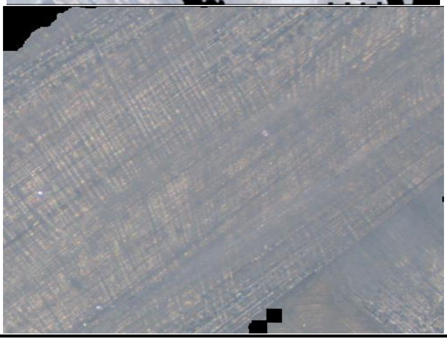

Table S4. 3D Topographical analysis for cured laminate surfaces as a function of POSS content following exposure to AO in simulated space conditions for a period of 12 months.

AO exposure	$1_{50}2_{50}3_{0}$	$1_{48}2_{48}3_{5}$
0 Months (before exposure)	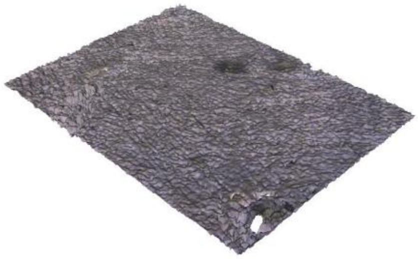	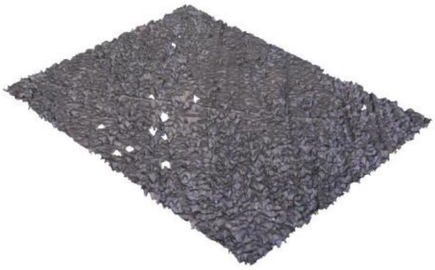
4 months	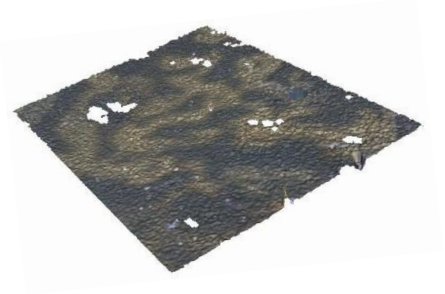	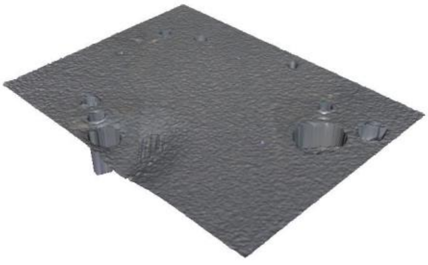
8 Months	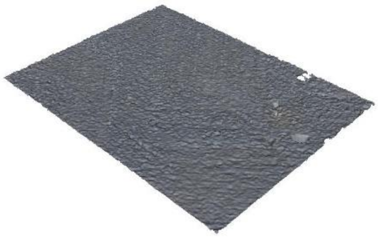	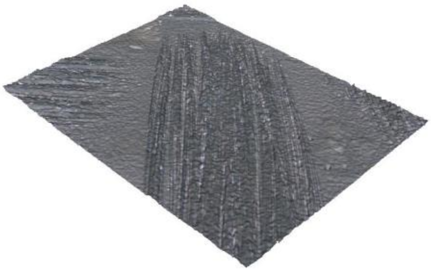
12 Months	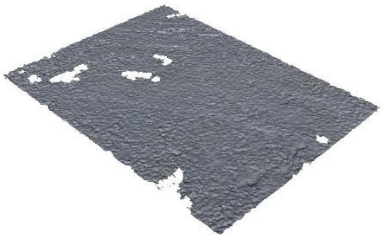	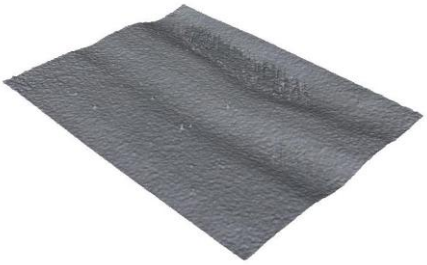
AO exposure	$1_{45}2_{45}3_{10}$	$1_{40}2_{40}3_{20}$
0 Months (before exposure)	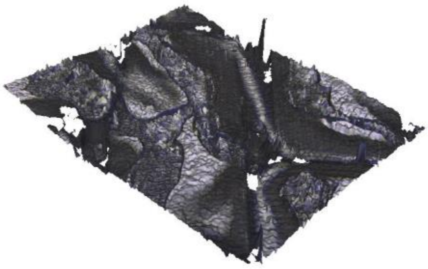	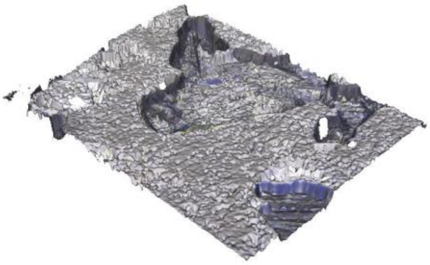
4 months	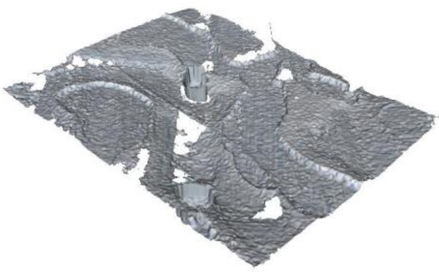	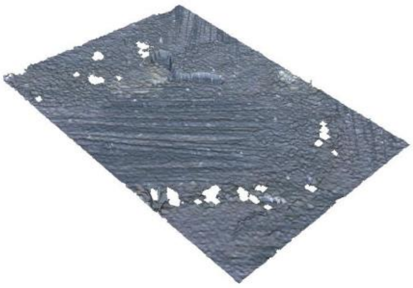

8 Months	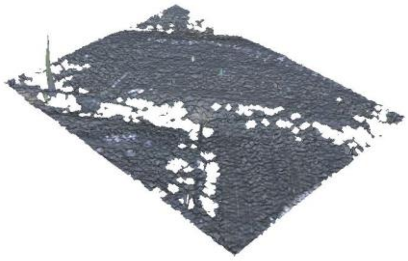	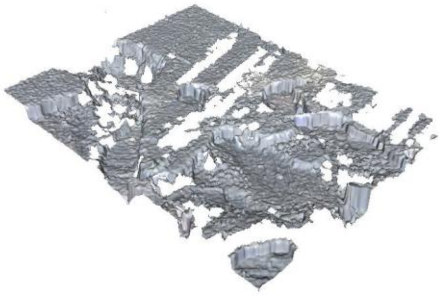
12 Months	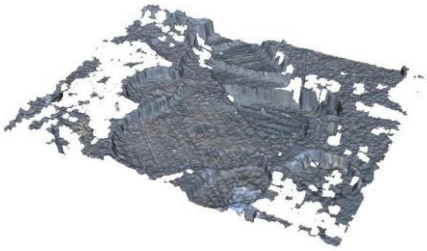	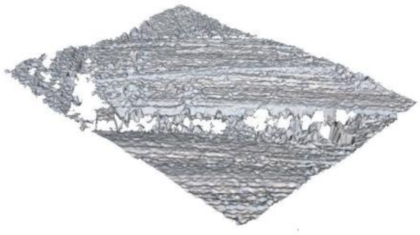

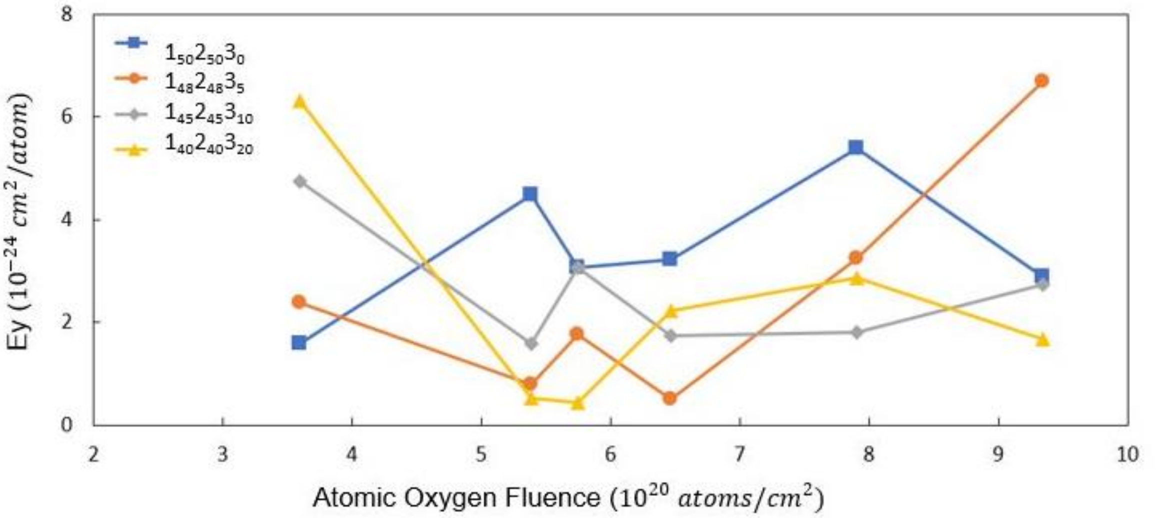

Figure S2. Erosion yield obtained for all the POSS content samples after exposure.

Table S5. Characteristic FTIR of the absorbance bands for the cured $1_{45}2_{45}3_{10}$ samples before and after 12 months of exposure in simulated LEO.

Wavenumber (cm^{-1})	Intensity	Functional Group
1100	Medium	POSS Cage Si-O-Si, asymmetric stretch
1450	Medium, Sharp	Aromatic ring, C=C stretch
1725	Strong, Sharp	Saturated carbonyl, C=O stretch
2850	Medium	Oxirane ring, C-H stretch
2920	Medium	Aliphatic amine, N-H stretch
3500	Strong, Broad	Secondary alcohol, O-H stretch
